# Profile of dog bite victims in Jos Plateau State, Nigeria: a review of dog bite records (2006-2008)

**DOI:** 10.11694/pamj.supp.2014.18.1.4341

**Published:** 2014-07-21

**Authors:** Olaniran Alabi, Patrick Nguku, Silvester Chukwukere, Ayika Gaddo, Peter Nsubuga, Joliath Umoh

**Affiliations:** 1 Nigeria Field Epidemiology and Laboratory Training Program, Nigeria; 2 National Veterinary Research Institute, Vom, Nigeria; 3 Evangelical Churches of West Africa Clinic, Jos, Nigeria; 4 Global Public Health Solutions, Decatur, Georgia, USA; 5 Ahmadu Bello University, Zaria, Nigeria

**Keywords:** Rabies, control, dog bites

## Abstract

**Introduction:**

Dogs are the major reservoir of rabies virus in Nigeria; transmission to humans is via a bite by rabid dog. Between 2006 and 2008 National Veterinary Research Institute (NVRI) rabies laboratory reported increased numbers of rabies in dogs and human dog bites. The objective of the study was to use veterinary and health records to develop a profile of bite victims and recommend appropriate public health actions.

**Methods:**

We used the dog brain specimen result register of Rabies Laboratory of NVRI, from “January, 2006” to “December, 2008” and traced dog bite cases. Structured questionnaires were administered to persons who reported dog bite incident and could be traced. We reviewed records from Evangelical Churches of West Africa (ECWA) clinic from “January, 2006” to “December, 2008” to collect detailed profiles of bite victims.

**Results:**

Bite victims linked to positive dog samples were traced to “ECWA clinic” from “January, 2006” to “December, 2008”. Most bite victims were <16 years 141 (72.3%), male 128 (65.6%), and 48.2% had primary school education. Bites were unprovoked 184 (94.4%), mostly on arms. 54.4% victims received complete post exposure prophylaxis (PEP). Majority of the biting dogs were housed and unvaccinated.

**Conclusion:**

This study provided important information on the profile of dog bite victims and highlights the need for a sustained awareness and education of children on the dangers of dog bite. It has shown lack of enforcement of regulations for licensing of dogs and rabies vaccination.

## Introduction

Dog bites pose a major public threat both in developed and developing nations. In addition to the severe physical trauma and potentially permanent disfiguring wounds sustained after a dog attack, dog bite victims are often burned with emotional and psychological trauma [1–3]. Dog bites in humans are a public health problem worldwide and they expose victims to many potential zoonoses [4]. Globally millions of people are bitten by dogs; for example in 1994 it was estimated that over 4.7 million dog bites occurred annually in the United States (U.S.) and approximately 800,000 persons required medical care, 44% of whom are <14 year old children[5]. There are reports of serious dog bites resulting in fatalities usually involving children, more than a dozen fatalities related to dog bites occur each year in several countries and most victims are children as reported in the United Kingdom, Belgium, the U.S. [5, 6], and the United Republic of Tanzania [7]. The most feared complication of dog bite is rabies, though not all dog bites result in rabies, however, in Africa and most developing countries where there are preponderance of unvaccinated dogs, every dog bite should be assessed for risk of rabies infection. This is because the pet dogs may have come in contact with a stray dog carrying rabies virus in the recent past. Rabies is a universal disease, estimated to be responsible for at least 55,000 human deaths annually, mainly in the developing countries of Africa and Asia [8, 9]. Rabies is acute, progressive and highly fatal (approximately 100% fatality). The incidence of human rabies is increasing in many countries; this is likely to be related in part to the rapid growth rate of dog populations, which in many parts of Africa exceeds that of human populations [9, 10]. Most of Africa and specially western and central African countries, notification of rabies is not mandatory, so epidemiological data are scarce [11]. This has resulted in inadequate control measures possibly due to under reporting, lack of follow up on victims of rabid dog bites, lack of data on the public health impact of the disease, and absence of programs for effective vaccination of dogs and control of stray dogs.

In Nigeria rabies is endemic, and dogs are the main reservoir of the virus [12], almost all the documented rabies cases have been associated with dog bites [13]. Dog bites are therefore an important public health issue [14]. There has been evidence that latent rabies exist--apparently healthy dogs were reported to have come down with rabies in Nigeria [15, 16]. During the period 2006 - 2008 there were reported increases in the number of confirmed rabies cases in dogs and the number of dog bite cases in most parts of the country. During this period there was also a reported problem of low vaccination coverage in dogs and lack of adequate information on the demographic profile of dog bite victims. There was also an absence of programs for the control of stray dogs and clinical records indicated that most human dog bite cases had incomplete doses of rabies post exposure prophylaxis (PEP). The indigenous people of Jos, Plateau state, in Central Nigeria are mainly farmers, with a very high dog population, partly due to the fact that most of the communities eat dog meat and from the records reviewed, the state reported the highest number of human dog bites cases and laboratory confirmed dog rabies during the period of “January, 2006” - “December, 2008”. In March, 2009 we decided to conduct a study using dog bite and veterinary records to develop a profile of dog bite victims in order to recommend appropriate public health actions to the authorities of Plateau state that can protect the population at risk from dog bites and rabies.

## Methods

**Study site:** We conducted the study in Jos, Plateau state, which is located in the central region of Nigeria. It is in the Guinea savannah zone with a population of over 3 million (according to the 2009, census). Jos had reported several dog bite cases to the Rabies Laboratory at the National Veterinary Research Institute, Vom, from 2006-2008.

**Data collection:** The standard practice is when a person is bitten by a dog that has no rabies vaccination history, such dog is killed and the head or brain is sent to the laboratory for rabies test. We used the dog brain specimen result register of the Rabies Laboratory of the National Veterinary Research Institute (NVRI). We reviewed records from “January, 2006” to “December, 2008” to obtain information about dog vaccination and ownership status and traced dog bite cases. We administered structured questionnaires to all persons who reported in the clinic for treatment of dog bite and could be traced. We also reviewed the records from Evangelical Churches of West Africa (ECWA) clinic from “January 2006” to “December, 2008” to collect information on age, sex, educational level, site of bite, and reason for bite, number of doses of PEP received and reasons for not completing the PEP. We selected this clinic because the NVRI laboratory records showed that all the bite victims during the period of study were advised to go there for the PEP.

**Data analysis:** Data from the study were entered into an Epi Info version 3.5.1 data base and we calculated frequencies, proportions, and percentages.

## Results

**Figure 1:**
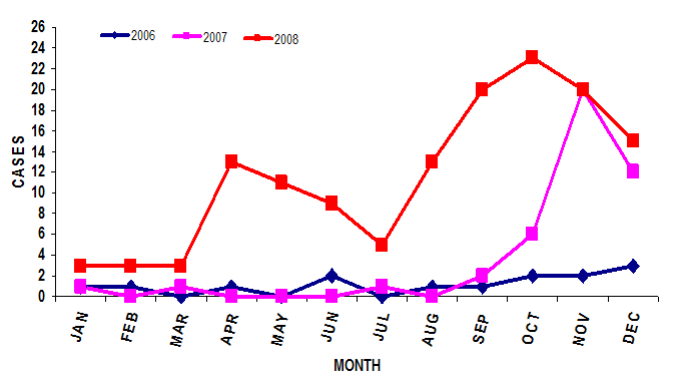
Monthly Trend of Dog Bite Cases in Jos, Plateau State, Nigeria, 2006-2008

A total of 554 dog specimens were submitted to the Rabies Laboratory following a bite of a human victim, of those 383 (69.1%) tested positive for rabies. However, only 195 (50.9%) of the 383 bite victims linked to a positive dog specimen could be traced. Consequently, a total of 195 structured questionnaires were administered to bite victims. About three quarters (141 (73%)) of the victims were aged <16 years; 128 (66%) were males; and 94 (48%) had primary level education. The majority, (165 (85%))of the bites were on the hands and only about half, (106 (54%)) of the victims took complete PEP (Table 1). For those who did not complete PEP, 64 (54%) reported it was too expensive while 15 (17%) reported that the vaccine was not available, We also found that 182 (93%) of the biting dogs were not vaccinated. A quarter, (51 (26%)) of the dogs were unrestricted, 151 (74%) had owners, and 152 (78%) of the biting dogs were aged between 18-24 months. Most, (184 (94%)) of the bites were reportedly unprovoked. The year 2008 recorded the highest number of dog bites with two peaks in April and October. In 2006 the number of dog bite cases was lowest. For all years the numbers of dog bite cases recorded were lowest at the beginning of the year and dog bites increased during the last 3 months (October-December) of the year (Figure 1).

**Table 1:** Profile of Dog Bite Victims in Jos, Plateau State from 2006-2008

Variables		Number N=195(%)
Age of Victims	0-< 16 years old	141 (72)
	16-30 years	30 (15)
	>30 years	24 (12)
Gender	Male	129 (66)
	Female	66 (34)
Current educational level	Primary education	126 (64)
	Secondary	48 (25)
	Tertiary	4 (2)
	No formal education	17 (9)
Site of bite	Arms	165 (85)
	Legs	21 (10)
	Shoulder	8 (4)
	Neck	1 (1)
Post exposure prophylaxis	Received 5 doses	106 (54)
	Received 3 doses	61 (31)
	Received 2 doses	19 (10)
	Received 0 dose	9 (5)

## Discussion

In our review of dog bite victims in Jos, Plateau State we found that male children <16 years in primary school were at greatest risk of being bitten by dogs. We also found a general increase in the number of dog bite cases towards the end of the year. Most of the bites were unprovoked and the bites were mainly on the hands. The biting dogs were adults, with owners, and the majority of the biting dogs were not vaccinated. Our findings of male children being at a greatest risk of dog bite are consistent with other reports of dog bites in children presenting to a pediatric emergency and trauma unit in the U.S. from 1995-1996 and in South Africa from 1991-2004 [3, 9, 17]. This could be because this age group may have limited experience or skills in recognizing a dog's body language and in perceiving hazardous situations that might trigger the occurrence of a biting incident [4]. Other reasons given were that children <15 years of age were more likely to provoke a dog or be playing with a dog. We also observed that most of the dog bites were on the hands, similar findings were reported in previous studies in Rome by Maragliano et al., in 2007 [18]. It is likely that this body region was used by the victims in protecting against dog attack [19]. However, studies in the U.S. and South Africa have reported that the common anatomical site for dog bite injuries in children >6 years is to the lower limbs [5, 20] this may be attributed to the short stature at this age. Our results showed that about three quarters of the dog heads tested were positive for rabies virus and most of the bites were unprovoked. This findings supports documented classical clinical signs exhibited by rabid dogs. Our results showed a general increase in the number of dog bite cases towards the end of the year; this finding is not in agreement with the findings of other studies [4], where more dog bites were recorded during the middle of the year (June -July). The possible explanation for these findings could be that, in addition to cultural issues of dog eating as food this time coincides with the holiday period for schools and festive season with generally very high human traffic. At this time there is also an increase in the number of dogs purchased and brought home in anticipation for slaughter. This increases the chances of dog human contact as most of the biting dogs had owners and but were unrestricted. This study was limited by incomplete data on dog bite victims, because of the inadequate accompanying information on the suspected rabies dog brains that were submitted to the Rabies Laboratory for diagnosis, thus we could not link all the bite victims to rabies positive dog heads and this made it impossible to trace all the dog bite cases to find out the outcome of the bite. Additionally because the scope of the study was limited to one facility, we cannot generalize these findings to the whole of Plateau state.

## Conclusion

The major conclusions from this study were that the profile dog bite victims were young males <16 years, dog bites were mostly on the hands, under unprovoked circumstances, by owned adult dogs which were unvaccinated. Almost half of bite victims did not complete PEP. Dog bites and dog rabies cases were highest towards the end of the year. We recommended that there should be strengthened collaboration between human and animal health authorities in rabies control, dog bite victims should be followed up to ensure completion of PEP in Plateau state. We also recommended that the health authorities should improve public health education on dangers of dog bites and the importance of dog vaccination, and responsible dog ownership, including restricted dog movements. The Government should ensure availability of vaccines at subsidized rates, intensify annual mass vaccination of dogs, remove stray/unrestricted dogs, and enforce leash laws. We believe these recommendations will go a long way in reducing dog bites and rabies in Plateau state and Nigeria. We recommend that dog bite victims should be followed up to ensure completion of PEP. There should be enforcement of regulations for licensing of dogs and responsible dog ownership. Government needs to ensure availability of vaccines and to intensify annual mass vaccination of dogs and remove unrestricted dogs.
